# Lance Adams Syndrome in the Setting of COVID-19 Pneumonia

**DOI:** 10.7759/cureus.60621

**Published:** 2024-05-19

**Authors:** Suparna R Krishnaiengar, Luis Cruz-Saavedra, Venkat Srikar Lavu, Ramon E Bautista

**Affiliations:** 1 Neurology, University of Florida College of Medicine – Jacksonville, Jacksonville, USA; 2 Neurology, University of Florida, Gainesville, USA

**Keywords:** post-hypoxic myoclonus, covid-19, #covid-19 respiratory failure, hypoxic ischemic brain injury, lance-adams syndrome (las)

## Abstract

Lance-Adams syndrome (LAS) is a rare clinical presentation of hypoxic-ischemic brain injury typically occurring in the setting of cardiac arrest. It is rare for it to be associated with respiratory failure. The advent of the COVID-19 pandemic heralded a new cause of respiratory failure, and not much is known about the occurrence of Lance-Adams syndrome in the context of COVID-19 pneumonia. A 23-year-old male was brought to the emergency department (ED) after being found unconscious at home. He had prominent generalized myoclonus in the context of COVID-19 pneumonia and a possible clonazepam overdose. Magnetic resonance imaging (MRI) of the brain with and without contrast revealed findings suggestive of hypoxic-ischemic brain injury. A diagnosis of LAS was made based on electroencephalography (EEG). As LAS typically carries a relatively favorable prognosis, aggressive treatment was pursued. This resulted in a fairly good outcome, although he had to be maintained on several antiseizure medications. Our case is a rare occurrence of Lance-Adams syndrome in the setting of respiratory failure and COVID-19 pneumonia in the absence of cardiac arrest. It is critical to distinguish myoclonic status epilepticus (MSE) from Lance-Adams syndrome due to the difference in prognosis. Our case can provide future direction for studies in a larger cohort of patients to see if LAS is frequently associated with respiratory failure secondary to COVID-19 pneumonia in the absence of cardiac arrest. It is important to consider Lance-Adams syndrome as one of the emerging neurological complications of COVID-19 pneumonia.

## Introduction

Hypoxic ischemic brain injury is often a result of cardiac arrest. This is true even when it is observed in the setting of the COVID-19 infection [1]. Other causes of hypoxic-ischemic brain injury that are less common include carbon monoxide poisoning, drug overdose, and head trauma [2-4]. Lance-Adams syndrome (LAS) is a rare clinical entity that was first described in 1963 and occurred in hypoxic-ischemic brain injury. Little is known about the possibility of respiratory failure, specifically COVID-19 pneumonia, causing hypoxic-ischemic brain injury. We present a case of Lance Adams syndrome in a patient with COVID-19 pneumonia in the absence of cardiac arrest.

## Case presentation

**Figure 1:**
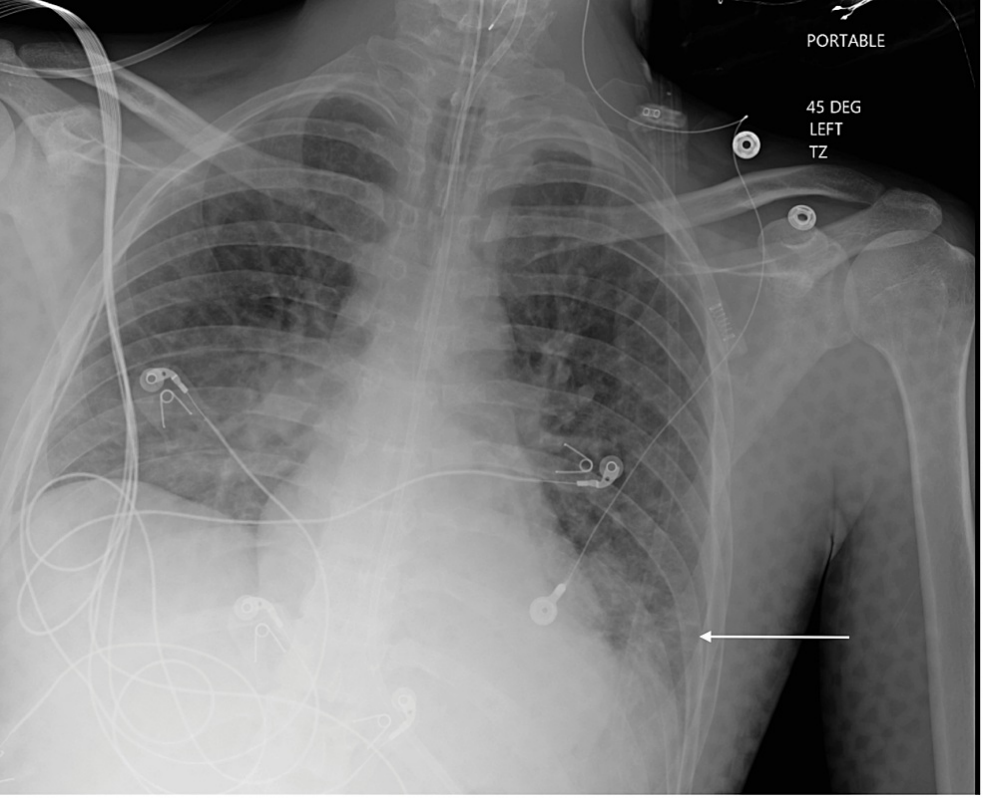
Chest X-ray revealed confluent airspace opacity in the left lower lobe likely representing pneumonia or atelectasis.

**Figure 2:**
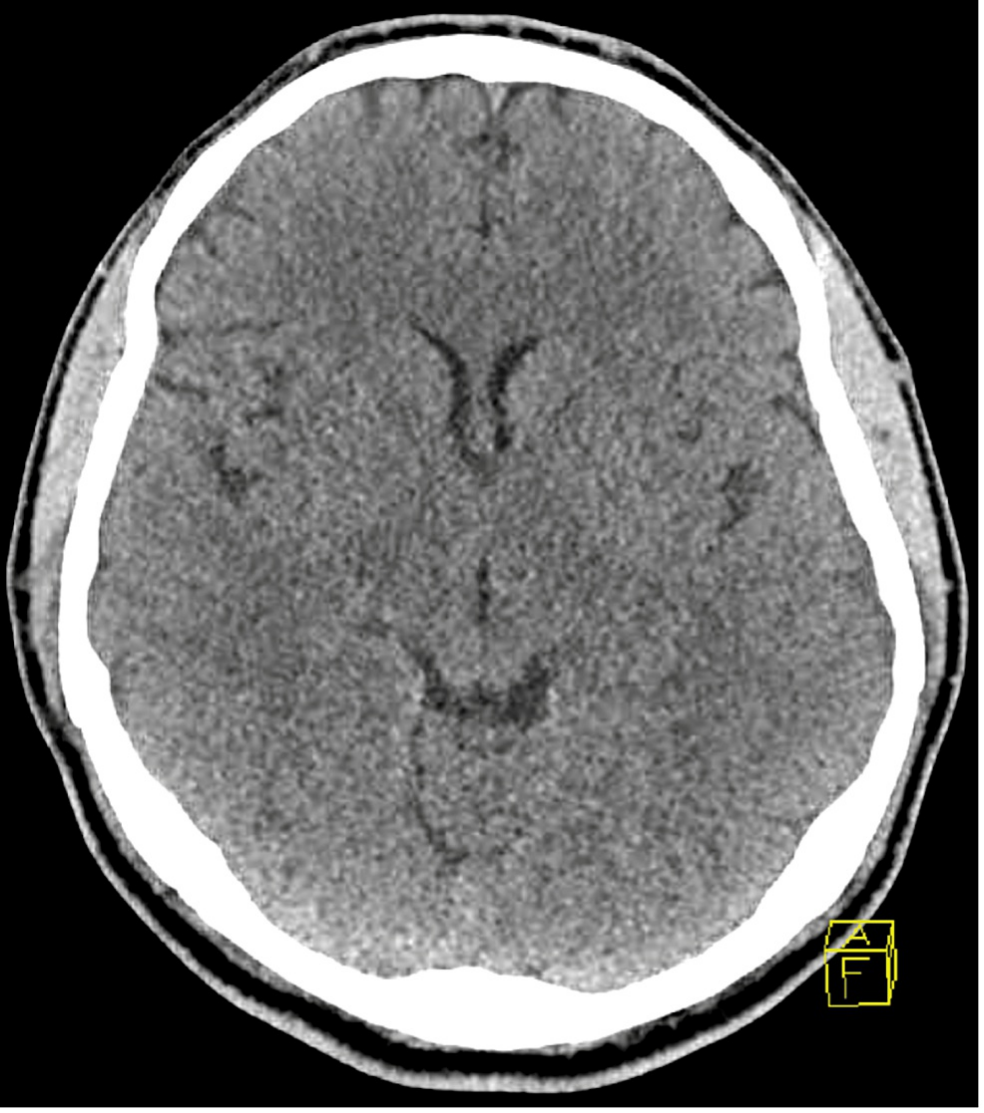
Computed tomography of the head did not indicate any intracranial abnormalities.

A 23-year-old male patient with a history of hemophilia B and depression was brought to the emergency department (ED) after being found unconscious at home for an unknown period of time. Emergency medical services administered naloxone without any clinical improvement. Prior to arrival in the ED, the airway was secured with a laryngeal tube, and the patient was subsequently intubated and maintained on mechanical ventilation. A clonazepam overdose was suspected but not confirmed. In the ED, his vital signs were stable other than tachycardia. Myoclonic jerks were noted in the presentation. He had a Glasgow Coma Scale score of 5 for eye-opening to pain, absent verbal response, and presence of extensor posturing. He developed a fever and was found to be positive for the SARS-CoV-2 infection. His chest X-ray revealed confluent airspace opacity in the left lower lobe, likely representing pneumonia or atelectasis (Figure 1). The computed tomography (CT) of the head did not indicate any intracranial abnormalities (Figure 2).

He exhibited brief, non-purposeful jerking of the bilateral upper and lower extremities and face, including the palatal myoclonus, aggravated by noxious stimuli. Clusters of these involuntary movements lasted more than five minutes, and he had multiple clusters per hour. He was admitted to the neurological intensive care unit (NICU) for further evaluation.

**Figure 3:**
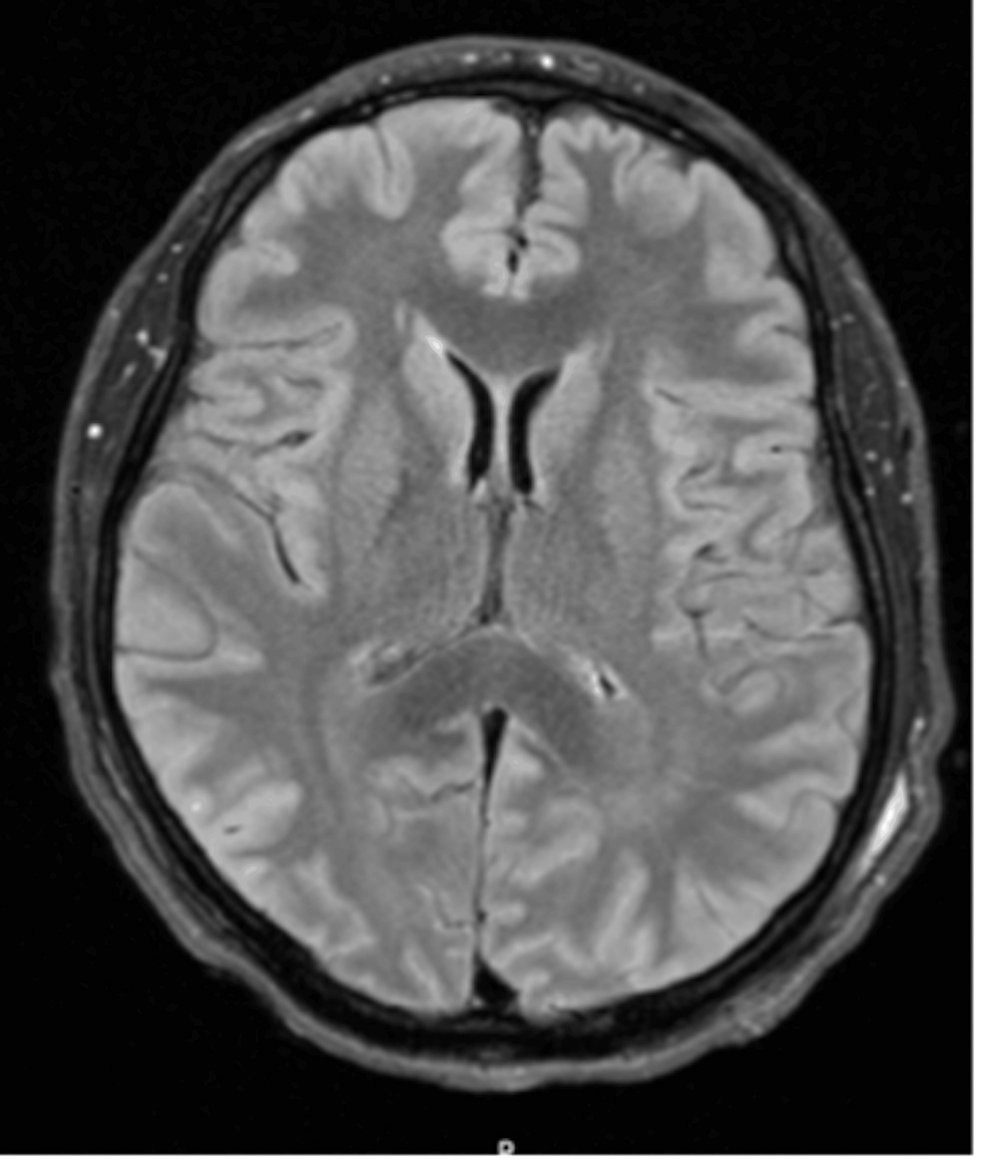
MRI brain T2-FLAIR sequence one day after admission was unremarkable.

**Figure 4:**
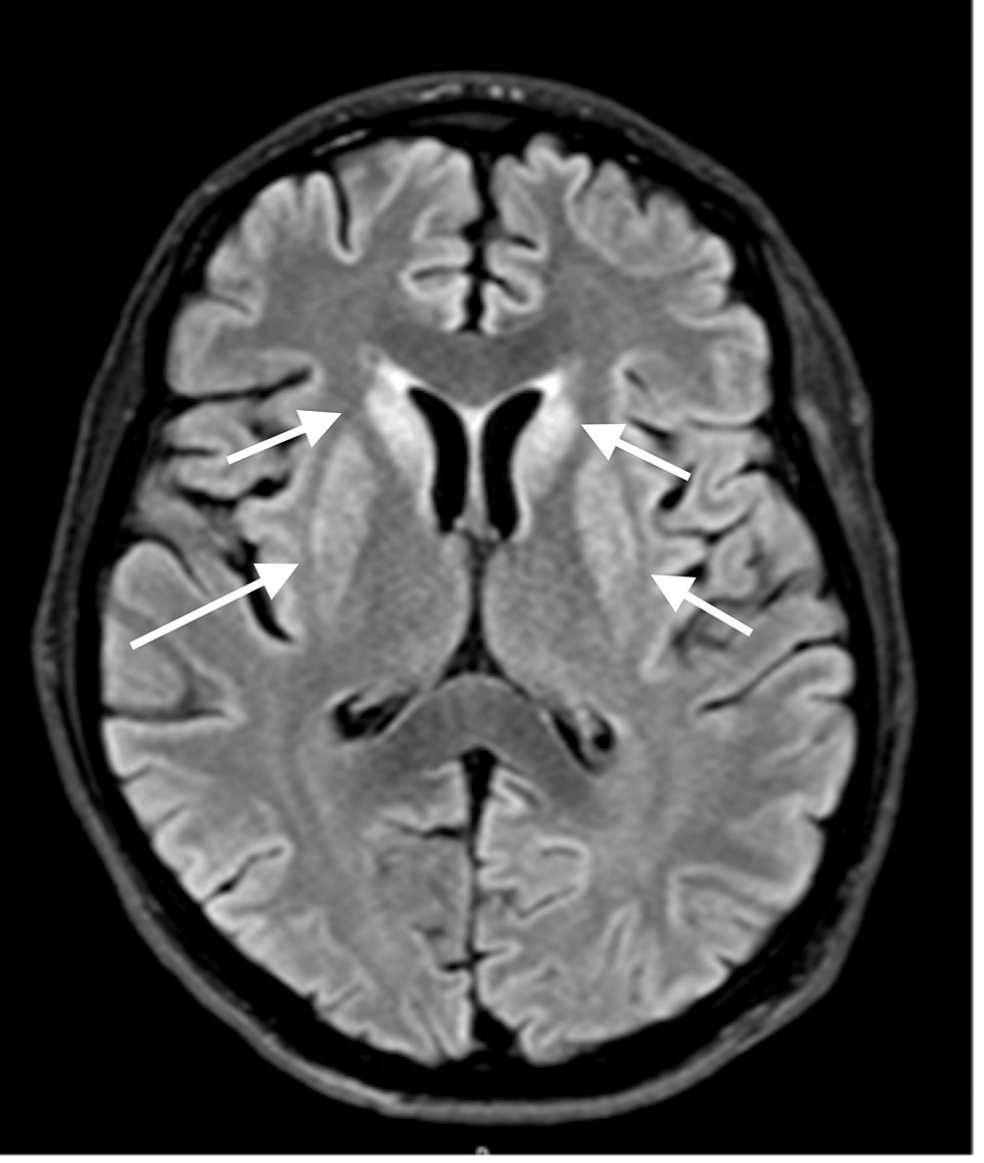
Repeat MRI of the brain with and without contrast, three weeks after admission, revealed T2-FLAIR hyperintensities in the bilateral basal ganglia, consistent with hypoxic ischemic brain injury.

**Figure 5:**
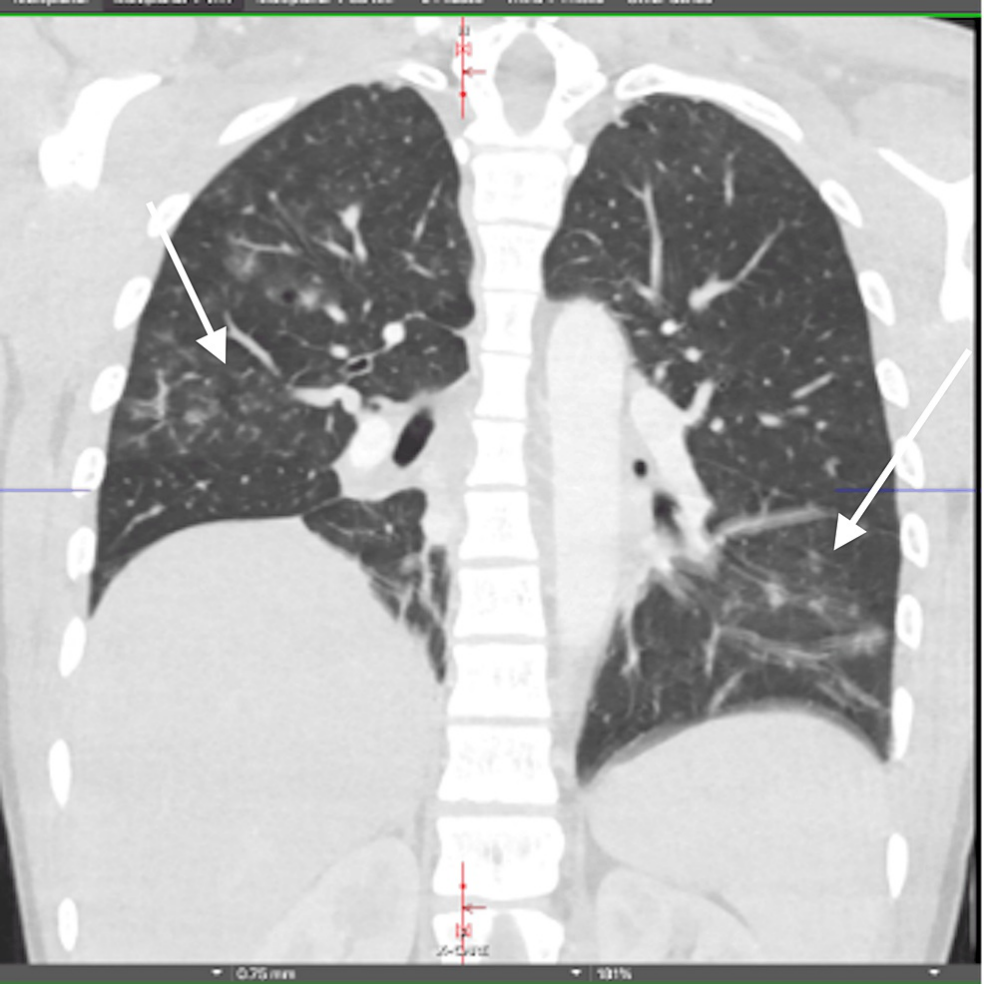
CT of the chest revealed bibasilar atelectasis and multifocal ground glass opacities within bilateral lungs, consistent with COVID-19 pneumonia.

As part of his workup while in the NICU, magnetic resonance imaging (MRI) of the brain with and without contrast was done one day after admission, which was unremarkable (Figure 3). However, a repeat MRI of the brain with and without contrast, three weeks after admission, revealed T2-FLAIR hyperintensities in the bilateral basal ganglia, consistent with hypoxic-ischemic brain injury (Figure 4). A CT of the chest performed two weeks after admission revealed bibasilar atelectasis and multifocal ground glass opacities within bilateral lungs, consistent with COVID-19 pneumonia (Figure 5).

**Figure 6:**
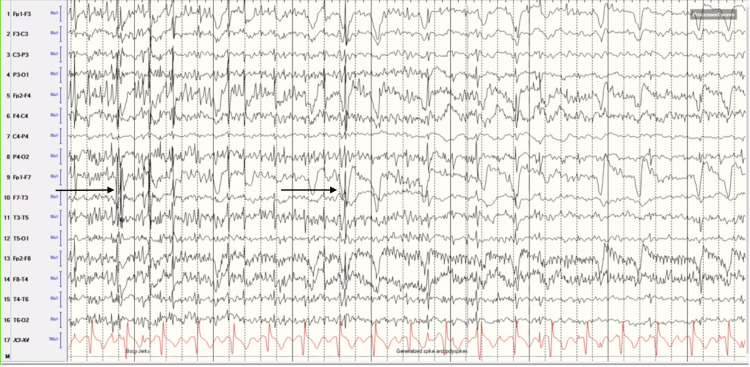
EEG revealed diffuse slowing and generalized spikes and polyspikes, in frequent runs at times, time locked to myoclonus.

On the day of admission, he was placed on long-term video electroencephalography (EEG) monitoring. The EEG revealed diffuse slowing and generalized spikes and polyspikes in frequent runs at times, time-locked to myoclonus, suggestive of Lance-Adams syndrome (Figure 6). As Lance Adams syndrome typically carries a relatively favorable prognosis, aggressive treatment was pursued.

He was started on high doses of valproic acid and levetiracetam, but despite aggressive medical therapy, attempts to wean sedation were unsuccessful, leading to the recurrence of generalized myoclonus. At this point, propofol was initiated and transitioned to midazolam. Subsequently, ketamine and pentobarbital were started to achieve pharmacologically induced coma with burst suppression on the EEG while optimizing his antiseizure medication regimen with high doses of lacosamide and primidone. After about 48 hours of burst suppression, he was successfully weaned from sedation. He was extubated two weeks after admission. Of note, throughout the course of his treatment, he did not receive cardiac resuscitation.

He was discharged from the hospital six weeks after his admission. At the time of discharge, he was alert and able to follow commands and carry on a simple conversation. He was able to move all his extremities, although he continued to experience myoclonus and required a gastric tube for feeding. His oxygen saturation in room air was normal, and he did not require any supplemental oxygen. He was discharged to an inpatient rehabilitation facility on valproic acid, lacosamide, levetiracetam, and primidone.

## Discussion

Our patient was positive for SARS-CoV-2 infection, and his chest imaging was suggestive of COVID-19 pneumonia. Interestingly, our patient did not have a cardiac arrest at any time. The respiratory failure, possibly due to COVID-19 pneumonia, could have contributed to the hypoxic-ischemic brain injury.

Previous cases with neurologic presentations in COVID-19 infection suggest possible mechanisms of brain pathology, such as viral invasion, inflammatory syndrome, and hypercoagulability. There is also evidence of hypoxic-ischemic encephalopathy associated with the COVID-19 infection. The basis of basal ganglia hyperintensities in the T2 sequence of brain MRI could be hypoxic-ischemic injury causing cytotoxic edema [5].

Myoclonic jerks are a marker for severe brain injury, and our patient had them early in his presentation. Post-hypoxic myoclonus can be either myoclonic status epilepticus (MSE), LAS, or subcortical myoclonus.

Typically, MSE is characterized by persistent bilaterally synchronous myoclonus of the face, limbs, and axial musculature, often with eye opening and upward deviation of the eyes. It typically begins within 72 hours after cardiac arrest and stops after a few days. MSE on the first day after primary circulatory arrest carries a poor prognosis [6]. It is often associated with poor outcomes, even with intact brainstem reflexes. Favorable prognostic indicators are intact cortical somatosensory evoked potential (SSEP), reactive EEG, and whether the circulatory arrest was secondary to respiratory failure.

LAS is a rare condition classically associated with action myoclonus on awakening from anoxic brain injury. In LAS, post-hypoxic myoclonus occurs days to weeks after successful cardiopulmonary resuscitation. In rare instances, it can occur on the first day after presentation, and myoclonus can be triggered by external stimuli or intentional actions, like in our patient [7]. Myoclonus is persistent in some cases [8].

It is important to accurately diagnose and distinguish between MSE and LAS, as the prognosis is different. Typically, MSE carries a poor prognosis, and LAS has a more favorable outcome. If a poor outcome is predicted incorrectly, it can result in premature withdrawal of care, and if there is an inaccurate optimistic prediction, it can lead to futile prolongation of life.

Other than some clinical characteristics, EEG can be useful in distinguishing between the two. EEG in MSE typically evolves in four stages. Initially, burst suppression with high-amplitude polyspikes is seen evolving into longer bursts and lower-amplitude polyspikes, followed by loss of amplitude and complexity, and finally generalized periodic discharges progressing to diffuse attenuation. Patients with this pattern of evolution typically do not show a favorable outcome [9]. 

EEG in LAS can be nonspecific, such as diffuse slowing. In some instances, it can be focal spikes or polyspikes with jerk-locking to spikes over the sensorimotor cortex or vertex preceding the jerk [10]. The EEG in our patient was suggestive of LAS, and expecting a favorable outcome, a decision was made to pursue aggressive medical therapy with pharmacologically induced coma and multiple anti-seizure medications.

The limitation to using EEG in distinguishing MSE from LAS is that EEG findings may be confounded by sedating medications that may induce burst suppression.

## Conclusions

With the onset of the COVID-19 pandemic, it is important to identify if hypoxic-ischemic brain injury may occur because of COVID-19 pneumonia-related respiratory failure. Our case was unique as the patient did not have a cardiac arrest, and the COVID-19 pneumonia likely contributed to the Lance Adams syndrome.

Distinguishing between LAS and MSE with the help of the EEG is important in determining aggressive treatment versus withdrawal of care, as clinical features between the two often overlap. Clinicians should consider obtaining brain imaging and EEG in patients with myoclonus associated with suspected hypoxic-ischemic brain injury. Further studies need to be done to evaluate the association between COVID-19 pneumonia-related respiratory failure and hypoxic-ischemic brain injury.
